# Falcarindiol Enhances Cisplatin Chemosensitivity of Hepatocellular Carcinoma *via* Down-Regulating the STAT3-Modulated PTTG1 Pathway

**DOI:** 10.3389/fphar.2021.656697

**Published:** 2021-05-07

**Authors:** Han Hong, Zhengkang Jin, Tao Qian, Xiaoyong Xu, Xiang Zhu, Qiang Fei, Jiamei Yang, Chengjun Sui, Minhui Xu

**Affiliations:** ^1^ Department of Hepato-Pancreato-Biliary Surgery, The Affiliated Suzhou Hospital of Nanjing Medical University, Suzhou, China; ^2^ Department of General Surgery, Affiliated Hospital of Integrated Traditional Chinese and Western Medicine, Nanjing University of Chinese Medicine, Nanjing, China; ^3^ Department of Special Treatment I and Liver Transplantation, Shanghai Eastern Hepatobiliary Surgery Hospital, Shanghai, China

**Keywords:** falcarindiol, hepatocellular carcinoma, cisplatin chemosensitivity, stat3, PTTG1

## Abstract

Hepatocellular carcinoma (HCC) is the most frequent primary liver malignancy globally and the third leading cause of cancer-related death. Chemotherapy is one of the main methods in treating HCC, while recent studies have found that the resistance of HCC to chemotherapeutic drugs reduces the efficacy of the chemotherapy. Falcarindiol (FAD) is a cytotoxic and anti-inflammatory polyacetylenic oxylipin found in food plants of the carrot family (Apiaceae), while its role in HCC remains to be explored. Here, HCC cells (Huh7 and LM3) were treated with FAD at different doses. Cell proliferation was tested by the cell counting kit-8 (CCK-8) method and colony formation assay, while the apoptosis was monitored by flow cytometry. The profiles of apoptosis-related proteins (Bax, bcl2, and Caspase-3), DNA repair proteins (Rad51, BRCA1, and MDC1), and the signal transducer and activator of transcription 3 (STAT3)/Pituitary Tumor Transforming Gene 1 (PTTG1) were verified by western blot (WB) or quantitative reverse transcription-polymerase chain reaction (qRT-PCR). The interaction between STAT3 and PTTG1 was verified by immunoprecipitation (IP). In addition, a xenograft tumor model was constructed in mice to explore the anti-tumor effects of FAD *in vivo*, and immunohistochemistry (IHC) was performed to count the number of Ki67-stained cells. As a result, FAD inhibited HCC cell proliferation and DNA repair, facilitated their apoptosis, and also enhanced cisplatin (DDP) chemosensitivity. The Combination Index (CI) evaluation showed that FAD and DDP had synergistic effects in repressing HCC cell proliferation. Besides, FAD dampened the STAT3/PTTG1 pathway expression. Further studies revealed that inhibiting STAT3 enhanced the inhibitive effect of FAD on HCC cells, whereas overexpressing PTTG1 attenuated the anti-tumor effect of FAD. Overall, our study illustrated that FAD is a potential anticancer drug and strengthens the chemosensitivity of HCC cells to DDP by inhibiting the STAT3/PTTG1 pathway.

## Introduction

Hepatocellular carcinoma (HCC) is among the most frequent malignancy worldwide. It is characterized by high incidence, high mortality, rapid growth, and strong invasiveness, which make it a serious threat to human health around the world (Liang et al., 2020). HCC is caused by multiple viral and non-viral factors such as hepatitis B virus (HBV) and hepatitis C virus (HCV) infection, chemical poisons, or non-alcoholic fatty liver disease (Li et al., 2020; Ragheb et al., 2020). Chemotherapy is commonly adopted in the HCC treatment, and high heterogeneity of the tumor often leads to reduced sensitivity to chemotherapy drugs, thus weakening drug efficacy and affecting therapeutic effects (Ragheb et al., 2020). Therefore, it’s urgent to explore effective anti-tumor drugs to improve the chemosensitivity of HCC.

Falcarindiol [FAD (3R, 8s)-falcarindiol, fadoh], a natural polyacetylene compound and a common natural flavonoid compound, has diverse biological activities, such as antioxidation and anti-inflammation (Venkatesan et al., 2018; Kobaek-Larsen et al., 2019). Recently, some reports have stated that FAD exerts a tumor-suppressive effect in multiple tumors, such as breast cancer (BC) (Lu et al., 2017). Also, FAD represses colon cancer cell growth in a xenograft tumor model, and it promotes the 5-fluorouracil-mediated anti-tumor effect (Jin et al., 2012). Even so, the specific effect by which FAD affects the chemosensitivity of HCC to cisplatin (DDP) needs to be further explored.

Signal transducer and activator of transcription 3 (STAT3) is a vital member of the STAT protein families, and numerous studies have confirmed that STAT3 accelerates cancer cell proliferation and invasion and induces chemoresistance (Lin et al., 2020). For instance, some studies have manifested that STAT3 targets oncogenes to enhance gastric cancer cell proliferation and metastasis (Ashrafizadeh et al., 2020). More importantly, targeting STAT3 has exhibited potent anti-tumor potential. It was discovered that miR-208b-5p inactivates epithelial-mesenchymal transition (EMT) by targeting the IL-9/STAT3 pathway and reducing the migration and invasion of non-small cell lung cancer (NSCLC) cells (Ma et al., 2020). What is more, Lee JH et al. found through a series of experiments that vitexin could be used as a potential blocking agent for the STAT3 signaling cascade to reduce the survival and invasion of HCC cells (Lee et al., 2020). On the other hand, pituitary tumor-transforming gene 1 (PTTG1) is a regulator of chromosome stability and is shown to aggravate cancer progression (Vlotides et al., 2007). For example, PTTG1 is overexpressed in laryngeal cancer, and predicts a poorer overall survival rate of patients with laryngeal cancer (Ma et al., 2020). Additionally, the dual-luciferase reporter assay showed that miR-655-3p dampens NSCLC cell migration and invasion by targeting PTTG1 (Wang et al., 2019). It is worth noting that PTTG1 is highly expressed in HCC, and inhibiting PTTG1 abates HCC cell proliferation and induces apoptosis (Liang et al., 2011). Interestingly, interleukin-6 (IL-6)-mediated STAT3 activation has been found to up-regulate PTTG1, thus leading to the malignant behaviors of prostate cancer cells (Huang et al., 2018). Nevertheless, it is not clear whether FAD influences HCC progression *via* STAT3/PTTG1 axis.

This study intends to explore the role and potential mechanism of FAD on HCC proliferation, apoptosis, and chemosensitivity to DDP. We found that FAD inhibited the proliferation of HCC cells and accelerated apoptosis. Moreover, FAD dose-dependently impeded STAT3 and PTTG1 expression. Hence, we conducted a series of experiments to confirm the effect of FAD on HCC to provide new theoretical references and insights for promoting the chemosensitivity of HCC.

## Methods

### Cell and Cell Culture

HCC cells (Huh7 and LM3) and human normal liver L-02 cells were bought from the Cell Center of the Chinese Academy of Sciences (Shanghai, China). They were then cultured in the RPMI1640 medium (Gibco; Thermo Fisher Scientific, Inc.) supplemented with 10% fetal bovine serum (FBS) (Gibco; Thermo Fisher Scientific, Inc.) and 1% penicillin/streptomycin (Beyotime, Shanghai, China) in an incubator at 37°C with 5% CO_2_. During the logarithmic growth phase, the cells were treated with 0.25% trypsin (Thermo Fisher HyClone, Utah, United States) and sub-cultured. Cells were treated with different doses of DDP (1–16 μg/mL), FAD (10–40 µM), or SC144 (0.6 μM) for 24 h. DDP (CAT. NO. S1166), FAD (CAT. NO. S3292), and the STAT3 inhibitor SC144 (CAT. NO. S7124) were bought from MC Selleck Chemicals (Shanghai, China).

### Cell Transfection

Huh7 and LM3 cells at the logarithmic growth stage were seeded in 6-well plates at 5 × 10^6^/well after trypsinization and sub-culture. GenePharma (Shanghai, China) provided the PTTG1 overexpression plasmids and the negative vector. Namely, the DNA fragments containing PTTG1 sequences were inserted into the plasmids. Huh7 and LM3 cells were transfected with the above vectors using Lipofectamine 3000 (Thermo Fisher, Shanghai, China) according to the manufacturer’s instructions. Quantitative reverse transcription-polymerase chain reaction (qRT-PCR) and western blot (WB) were adopted to examine whether PTTG was overexpressed. The cells were incubated at 37°C with 5% CO_2_ for 24 h.

### Colony Formation Assay

Huh7 and LM3 cells in the logarithmic growth phase were trypsinized with 0.25% trypsin, seeded into 30 mm culture dishes (100 cells/dish), and incubated at 37°C with 5% CO_2_ and saturated humidity for 14 days. When the cell colonies were visible, the supernatant was discarded and washed with PBS twice. Then, 4% paraformaldehyde was added for immobilization for 15 min. After immersion in PBS two times, an appropriate amount of crystal violet staining solution (Wuhan Google Biology Co., Ltd.) was added and maintained for 10 min. Next, the staining solution was washed away with running water. After drying, the number of colonies was counted by the ImageJ software.

### Cell Counting Kit-8 Experiment

The transfected Huh7 and LM3 cells in the logarithmic growth phase were obtained, trypsinized, and adjusted to reach a density of 2 × 10^3^ cells/mL. They were then inoculated in 96-well plates (100 μL cell suspension/well), and three repetitive wells were set in each group. The 96-well plates were then incubated. After 24 h, each well was supplemented with 10 μL cell counting kit-8 (CCK-8) solution (Beyotime, Shanghai, China) and incubated for another 1 h. After the culture, the 96-well plates were placed in a microplate reader to determine the optical density (OD) at 450 nm. After that, the OD value was observed at the 24th, 48th, and 72 nd h.

### Combination Index Evaluation

The HCC cells (including Huh7 and LM-3) were treated with different doses of cisplatin (1–16 μg/mL) with or without FAD (5–80 µM). The combination index (CI) was calculated using Calcusyn software (Biosoft, Cambridge, United Kingdom) to confirm whether FAD and DDP have a combination effect (Tao et al., 2020). The formulas are the following: fa = 1—(OD experiment/OD control). fa indicates cell inhibition rate. CI = (D)1/(Dx)1 + (D)2/(Dx)2 (Dx)1 and (Dx)2 are the concentrations of the single drugs required to produce an X% effect, and (D)1 and (D)2 are the drug concentrations in combination treatments, which produce the same X% effect. When CI < 1, FAD and DDP have a synergistic action, CI > 1 means one drug antagonizes another.

### Flow Cytometry

Huh7 and LM3 cells were trypsinized and collected through centrifugation (1500 RPM, 3 min). The obtained cells were operated following the Annexin V-FITC Apoptosis Staining/Detection Kit (ab14085, Abcam) instructions. Namely, the cells were cleared with PBS twice, and 400 μL pre-cooled PBS was added, and then 10 μL AnnexinV-FITC and 5 μL PI were supplemented. Afterward, they were incubated at 4°C in the dark for 30 min, and then the apoptosis was measured by BD FACSCalibur flow cytometer (Accuri C6, BD Biosciences, San Jose, CA, United States) immediately. The percentage of apoptotic cells was calculated by FlowJo software (Version 10, Tree Star, Ashland, OR, United States).

### Western Blot

Huh7 and LM3 were transfected, and the primary medium was discarded. The cells were then lyzed with the RIPA (containing 1% PMSF) lysis buffer and harvested through low-speed centrifugation (14,000 RPM, 30 min at 4°C) to extract the total protein. Afterward, the protein quantification was performed using the Bradford method with Bradford Protein Assay Kit (Beyotime, Shanghai, China), and the protein was boiled for 5 min, cooled on ice, and centrifuged for 30 s. Subsequently, the supernatant was retained for polyacrylamide gel electrophoresis, and the protein was transferred to the polyvinylidene fluoride (PVDF) membranes at 100 V for 1 h. Next, the membranes were blocked with 5% skimmed milk at room temperature (RT) for 1 h and incubated with the primary Anti-PTTG1 antibody (1:1000, ab79546), Anti-STAT3 antibody (1:1000, ab68153), Anti-p-STAT3 antibody (1:2000, ab76315), Anti-Bax antibody (1:1000, ab32503), Anti-bcl2 antibody (1:1000, ab32124), Anti-Caspase-3 antibody (1:1000, ab13847), Anti-RAD51 antibody (1:1500, ab133534), Anti-BRCA1 antibody (1:1000, ab238983), Anti-MDC1 antibody (1:1000, ab11171) and Anti-β-actin antibody (1:1000, ab8226) overnight at 4°C. After that, the membranes were rinsed with TBST twice and incubated with the luciferin-labeled secondary antibodies of Goat Anti-Rabbit (1:2500, ab6721) at RT for 1 h and washed three times. Finally, the membranes were exposed with ECL chromogenic agent (and imaged with a membrane scanner. The above antibodies were bought from Abcam (MA, United States).

### Quantitative Reverse Transcription-Polymerase Chain Reaction

The TRIzol reagent (Invitrogen, Carlsbad, CA, United States) was adopted to isolate the total RNA of Huh7 and LM3 cells. After the purity test, the total RNA was reversely transcribed into cDNA with the RevertAid First Strand cDNA Synthesis Kit (Thermo Fisher Scientific, Waltham, MA, United States). The cDNA synthesis conditions were 37°C for 40 min and 85°C for 5 s. The Rad51, BRCA1, MDC1, PTTG1, and STAT3-specific primers (Sangon Biotech, Shanghai, China) were applied for PCR using the SYBRGreen method, with cDNA as a template. PCR was completed by 40 cycles under 95°C for 30 s, 95°C for 5 s, 60°C for 30 s, and 73°C for 10 s. GAPDH served as the endogenous control of the above genes, and the relative gene expression was presented with the 2^−ΔΔCT^ method. The primer sequences were shown in Table 1.

**TABLE 1:** The primer sequences.

Gene name	Primer sequences (5^’^→3^’^)
PTTG1	F: TTT​AGG​TAA​GGC​TGG​TGG​GG
	R: CTT​CTC​CCA​CCT​TCC​CCA​AT
STAT3	F: CTG​AGG​AAA​CGC​TGA​CAT​CG
	R: CAC​TTC​CTG​TCT​GCC​ACC​TA
Rad51	F: GGT​CTC​TCT​GGC​AGT​GAT​GT
	R: TCT​GTT​CTG​TAA​AGG​GCG​GT
BRCA1	F: TGA​AGA​AAG​AGG​AAC​GGG​CT
	R: TGG​CTC​CCA​TGC​TGT​TCT​AA
MDC1	F: CAC​CCC​AAA​AGC​CCC​TTA​AC
	R: AGG​TAG​CTG​GAA​AGG​GTG​TC
GAPDH	F: CTC​CTC​CTG​TTC​GAC​AGT​CAG​C
	R: CCC​AAT​ACG​ACC​AAA​TCC​GTT

### Immunoprecipitation Experiment

After pEGFP-PTTG1-minigene plasmids were trypsinized with Xho, the linearized plasmid was homologous with the 12×MS2 sequence to construct the pEGFP-PTTG1-minigene-12×MS2 plasmid. Then, 10 μg pMS2-GFP and pEGFP-PTTG1-minigene-12×MS2 plasmids were co-transfected into Huh7 and LM3 cells, and the nuclear proteins of HCC cells were extracted 48 h later. Then, 3 mg nuclear protein was purified with 50 μL protein G-agarose (Santa Cruz, United States) at 4°C for 2 h. Afterward, the supernatant was maintained with the GFP antibody at 4°C overnight for shaking incubation. Then, 50 μL protein A/G-agarose was added and incubated for 2 h. Finally, the beads were washed and re-suspended in 50 μM 12×SDS-PAGE loading buffer and boiled for 10 min for the WB.

### Tumor-Bearing Model in Nude Mice

The experiment was authorized by the Animal Ethics Committee of the Affiliated Suzhou Hospital of Nanjing Medical University [approval number: SCXK(SU)2016-0002]. All experimental animals were bought from the Experimental Animal Center of Nanjing Medical University. Forty male nude mice (body weight: 20–22 g) aged 5–6 weeks were selected and raised under aseptic conditions. LM3 cell lines in the logarithmic growth phase were adjusted to a concentration of 1 × 10^7^ mL^−1^. Then, 0.1 mL cell suspension was injected subcutaneously into the left forearm axilla of the nude mice. The 40 nude mice were randomly divided into four groups, including the control group (only the same volume of PBS was injected), the DDP (2 mg/kg body weight) group, the DDP (2 mg/kg body weight) +FAD (10 mmol/kg body weight), and the DDP (2 mg/kg body weight) +FAD (20 mmol/kg body weight). The mice were intraperitoneally injected with cisplatin (2 mg/kg) every 4 days for 28 days and/or FAD (10–20 mmol/kg) every other day for 28 days. After the treatment with different factors, the mice were further raised under aseptic conditions. Four weeks later, all the animals were sacrificed by receiving anesthesia with phenobarbital sodium (30 mg/body weight), and the tumor tissues were completely removed. At week 2, 4, and 6, the exfoliated tumors were weighed separately, and the long diameter (a) and short diameter (b) of the tumors were accurately measured with the vernier caliper three times, and the mean value was taken to calculate the tumor volume, which was presented as 0.5 × a × b^2^.

### Immunohistochemistry

The tumor tissues exfoliated from the dead mice were paraffin-embedded, sectioned (4 μm), dewaxed with xylene, and hydrated with gradient alcohol. Then, the endogenous peroxidase was inactivated by blocking the sections with 3% H_2_O_2_ for 10 min, and the microwave repair was made with 0.01 mol/L sodium citrate buffer (pH = 6.0, 15 min). After that, the sections were blocked with 5% bovine serum albumin (BSA) for 20 min and incubated with the primary Anti-Ki67 antibody (1:1000, ab15580) at 4°C overnight. The next day, Goat Anti-Rabbit IgG (1:2500, ab6721) was added and incubated at RT for 20 min. After the sections were cleared with PBS, DAB was used for color development. The staining was observed under a microscope (BX50F; Olympus, Tokyo, Japan). The above antibodies were purchased from Abcam (MA, United States).

### Statistical Analysis

Experimental data were analyzed by SPSS18.0 statistical software (SPSS Inc., Chicago, IL, United States) and GraphPad Prism 8 (GraphPad Software, United States). The difference between the two groups was compared by student’s *t* test, and one‐way ANOVA was used for the analysis of multiple group data. *p* < 0.05 was considered to have statistical significance. The quantitative data were shown as the mean and SD values of independent experiments that were repeated at least three times.

## Results

### Falcarindiol Reduced Hepatocellular Carcinoma Cell Proliferation and Induced Apoptosis

**FIGURE 1:**
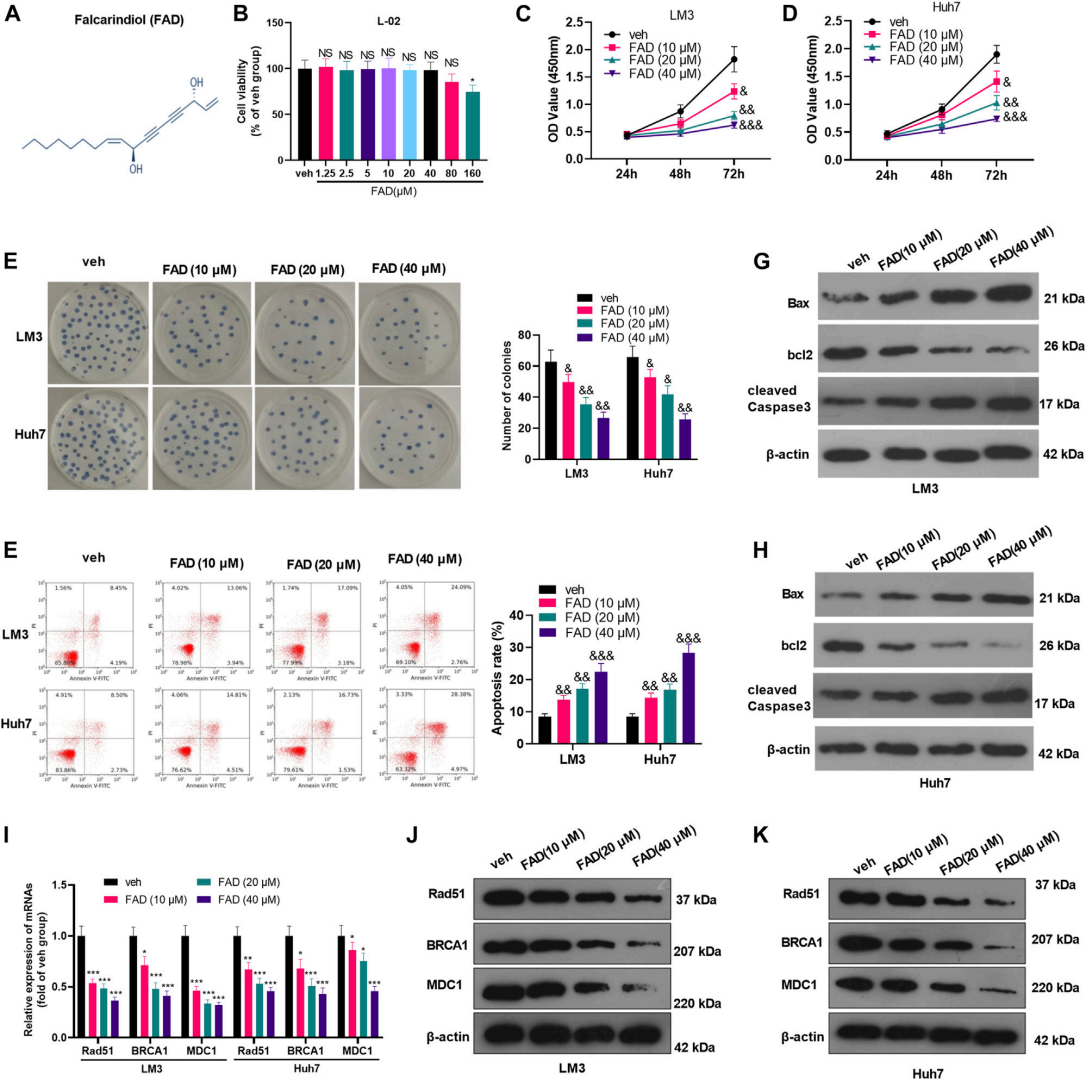
FAD reduced the proliferation and induced apoptosis of HCC cells **(A)**. The chemical structure of FAD was shown. **(B)**. Human normal liver L-02 cells were treated with different doses of FAD (0–160 μM) for 24 h, and CCK-8 assay was used to test cell viability, NS, * represents *p* > 0.05, *p* < 0.05, respectively. C-E. HCC cells (including Huh7 and LM3) were treated with 10–40 μM of FAD for 24 h. The proliferation of HCC cells was evaluated by CCK-8 assay **(C–D)** and colony formation assay **(E)**. **(F)**. FCM was carried out to test cell apoptosis **(G–H)**. WB was performed to determine the apoptosis-related proteins (including Bax, bcl2, and cleaved Caspase3) of HCC cells. &, &&, &&& represents *p* < 0.05, *p* < 0.01, *p* < 0.001, respectively. N = 3. I-K. qRT-PCR **(I)** and WB **(J–K)** were used for detecting DNA repair-related genes/proteins (including Rad51, BRCA1, and MDC1) in Huh7 and LM3 treated with 10–40 μM of FAD for 24 h *, **, *** represents *p* < 0.05, *p* < 0.01, *p* < 0.001, respectively. N = 3.

The chemical structure of FAD was shown in Figure 1A. To ensure the cytotoxic effect of FAD on normal liver cells, we treated human normal liver L-02 cells with different doses of FAD (0–160 μM) for 24 h. CCK-8 results showed that FAD had no significant effect on L-02 cell viability until its concentration reached 160 μM (Figure 1B). Thus, we treated HCC cells with 10–40 μM of FAD in the later experiment. HCC cell proliferation was evaluated by the CCK-8 assay and colony formation assay. As a result, FAD hampered HCC cell proliferation and colony formation (Figures 1C–E). Subsequently, flow cytometry (FCM) and WB were performed to determine the apoptosis of HCC cells. We found that FAD treatment increased HCC cell apoptosis dose-dependently (Figure 1F). Moreover, FAD enhanced Bax and cleaved Caspase3 expression in HCC cells while reduced the bcl2 level (vs. the veh. group) (Figures 1G,H). As DNA repair is a vital process of tumor cells against chemotherapy (Li et al., 2016; Chen et al., 2021), we then performed qRT-PCR to detect three DNA repair-related genes and proteins. It was found that FAD dose-dependently reduced the mRNA and protein levels of Rad51, BRCA1, and MDC1 (*p* < 0.05 vs. the veh group, Figure 1I–K). Therefore, we confirmed that FAD exerts anti-proliferative and pro-apoptotic effects in HCC cells.

### Falcarindiol Enhanced the Chemosensitivity of Hepatocellular Carcinoma to DDP

**FIGURE 2:**
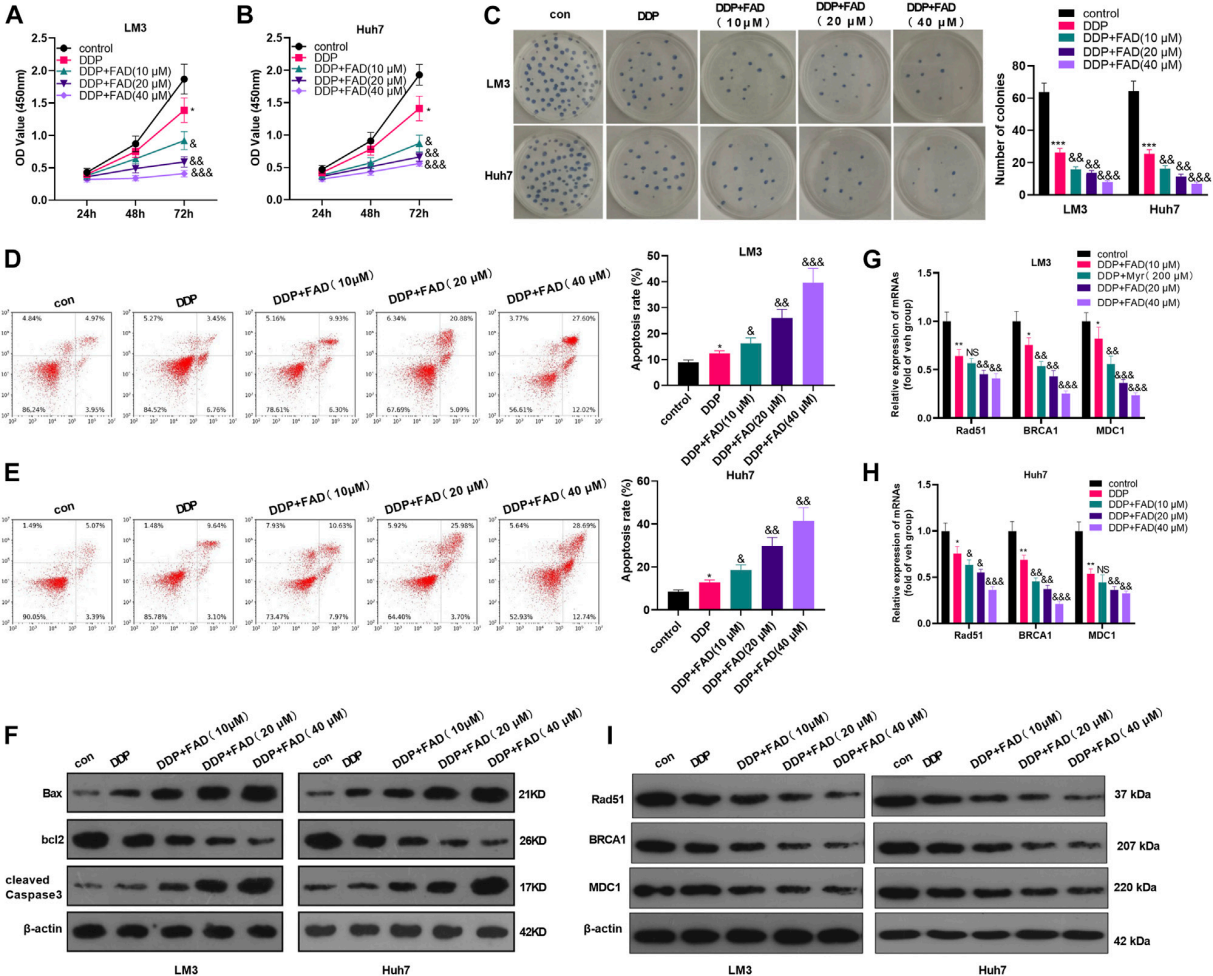
FAD enhanced the chemosensitivity of HCC to DDP. HCC cell lines (Huh7 and LM3) were treated with DDP (2 μg/mL) for 24 h. On this basis, FAD (10, 20, 40 µM) was applied to deal with the cells for 24 h. **(A–C)**, Cell proliferation was examined by CCK-8 assay and colony formation assay. **(D, E)**, Cell apoptosis was determined by FCM. **(F)**. WB was implemented to verify the expression of the apoptosis-related proteins (including Bax, bcl2, and cleaved Caspase3). G-I. qRT-PCR **(G–H)** and WB **(I)** were used for detecting DNA repair-related genes/proteins (including Rad51, BRCA1, and MDC1) in Huh7 and LM3 treated with FAD or for 24 h *, **, *** represents *p* < 0.05, *p* < 0.01, *p* < 0.001 (vs. con group), respectively. NS, &, &&, &&& represents *p* < 0.05, *p* < 0.01, *p* < 0.001 (vs. DDP group), respectively. N = 3. All experiments were repeated for 3 times.

**FIGURE 3:**
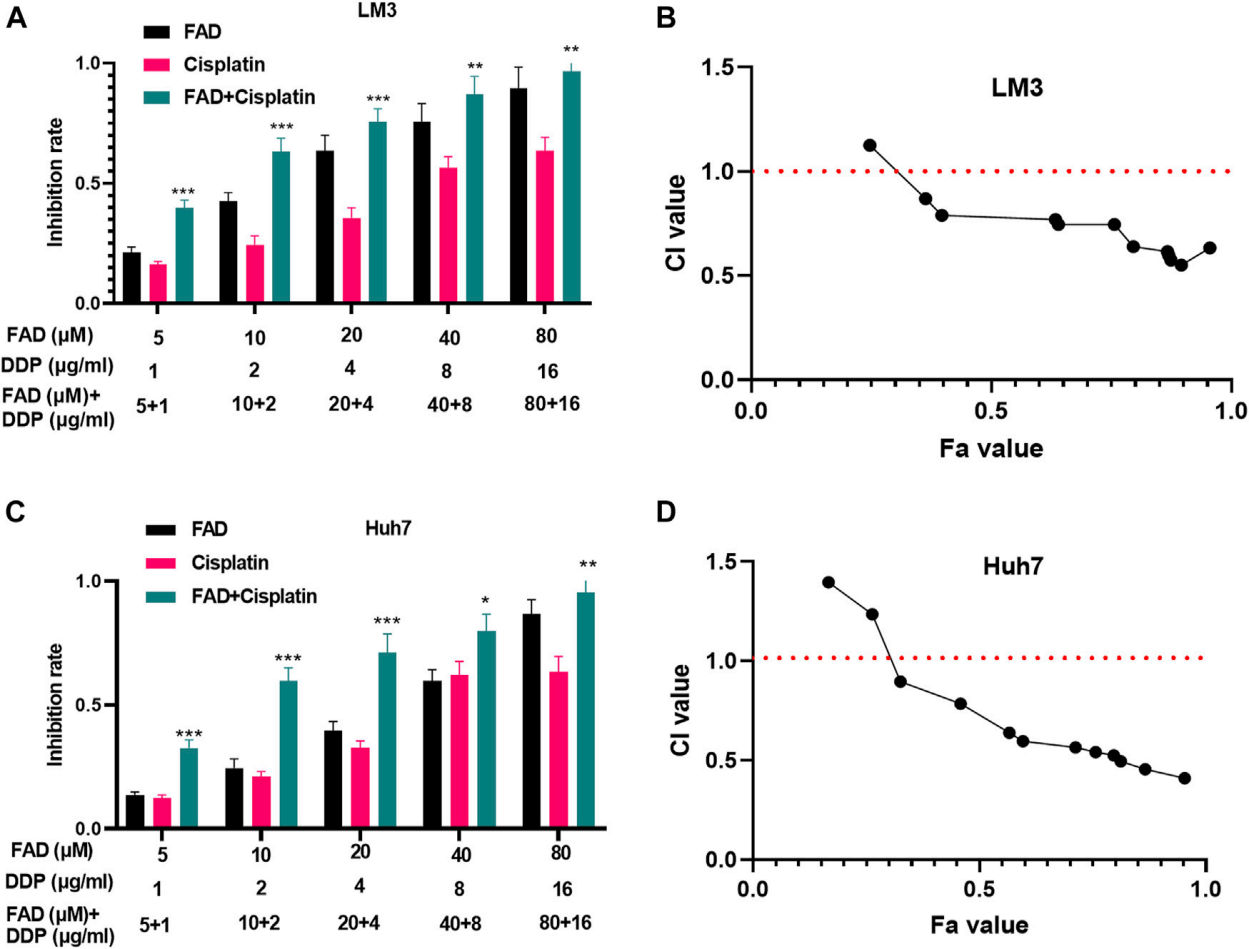
FAD had a synergistic effect with DDP in HCC cells. HCC cell lines (Huh7 and LM3) were treated with FAD (5, 10, 20, and 40.80 µM) with or without DDP (1, 2,4, 8, and 16 μg) for 24 h. **(A, C)**. The inhibition rate of Huh7 and LM3 cells under FAD/DDP treatment was measured *via* CCK8 assay. *, **, *** represents *p* < 0.05, *p* < 0.01, *p* < 0.001 (vs. DDP group), respectively. **(B, D)**. The synergistic effect of FAD and DDP were measured using the Median-effect principle, and the fa-CI curve graph of the two cell lines was shown. N = 3. All experiments were repeated three times.

Huh7 and LM3 cells were treated with DDP (2 μg/mL) and FAD (0, 10, 20, and 40 µM) for 24 h, respectively, to probe the impact of FAD on HCC chemosensitivity to DDP. The experimental results of the CCK-8 method and colony formation assay illustrated that DDP attenuated HCC cell proliferation. FAD enhanced the inhibition of DDP on HCC cell proliferation, and FAD with higher doses had more significant effects (*p* < 0.05 vs. DDP group, Figures 2A–C). Besides, FCM was implemented to test the apoptosis after the drug treatment. DDP promoted the apoptotic level of HCC cells, and FAD enhanced the pro-apoptotic effect of DDP on HCC cells (*p* < 0.05 vs. DDP group, Figures 2D,E). Moreover, WB proved that FAD promoted the profiles of pro-apoptotic proteins Bax and cleaved Caspase3 and inhibited the level of anti-apoptotic protein bcl2 compared with that of DDP treatment alone (Figure 2F). Then DNA repair-related genes and proteins (including Rad51, BRCA1, and MDC1) were detected in HCC cells. The qRT-PCR and WB results indicated that DDP reduced those genes and proteins, which were further inhabited with FAD treatments (*p* < 0.05 vs. DDP group, Figure 2G–I). Since FAD treatment in combination with DDP repressed HCC cells’ proliferation and promoted apoptosis, we further evaluated the CI of FAD and DDP on HCC cells. As the data showed, the combination of FAD and DDP enhanced DDP-induced anti-proliferative effect in two HCC cell lines (Figures 3A,C), and they had a synergistic effect (CI < 1) when the fa value was between 0.3 and 1 (Figures 3B,D). Collectively, FAD promoted DDP-induced anti-tumor functions in HCC.

### Falcarindiol Dampened the Expression of the STAT3/PTTG1 Signaling Pathway

**FIGURE 4:**
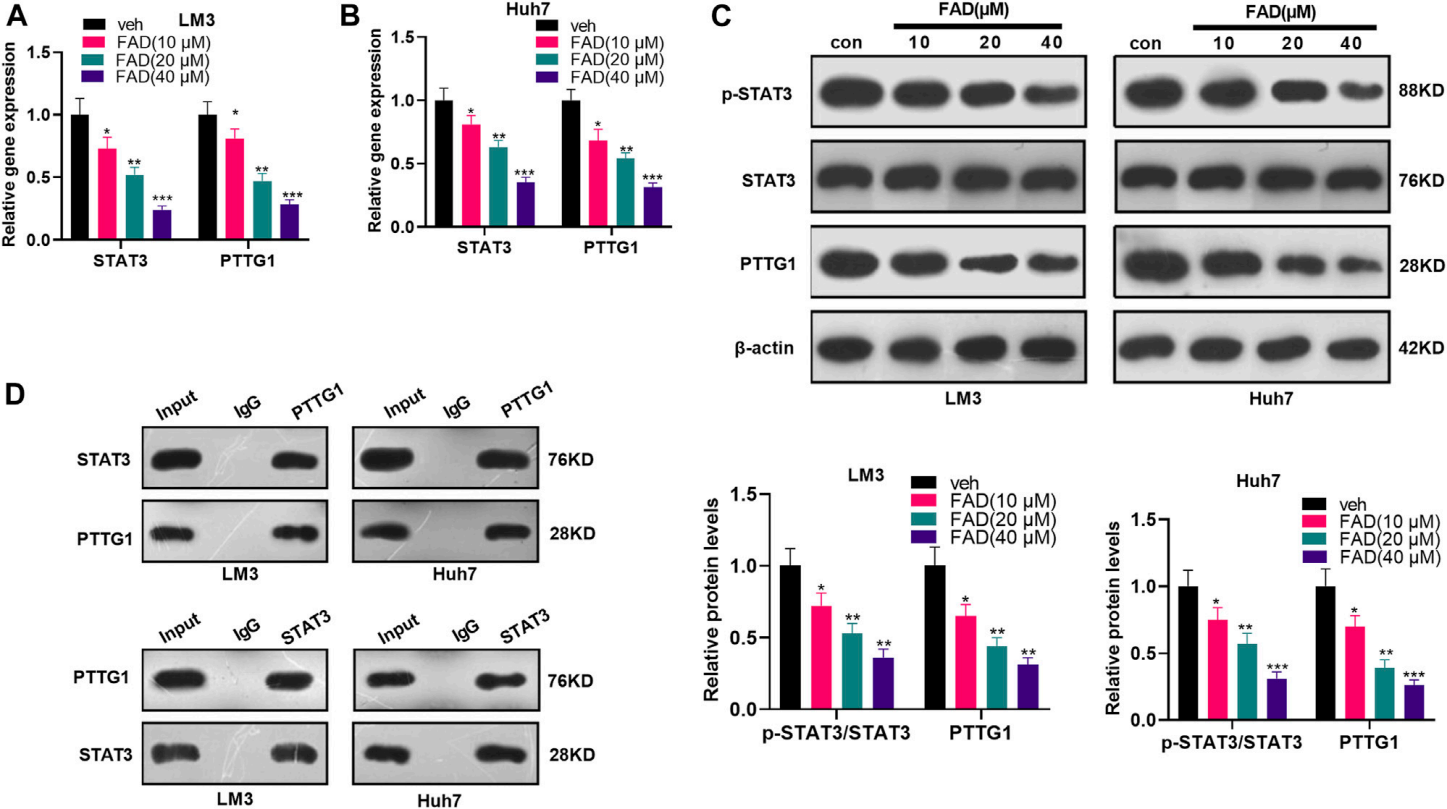
FAD inhibited the STAT3/PTTG1 expression. HCC cell lines (Huh7 and LM3) were treated with FAD (10, 20, and 40 µM) for 24 h. **(A, B)**. The STAT3/PTTG1 level was monitored by qRT-PCR. **(C)**. The STAT3/PTTG1 profile was determined by the WB. **(D)**. IP experiment was applied to clarify the binding association between STAT3 and PTTG1. *p < 0.05, **p < 0.01, ***p < 0.001 (vs. veh group). N = 3.

First, HCC cells were treated with FAD at different doses to investigate the effect of FAD on the STAT3/PTTG1 pathway. qRT-PCR illustrated that FAD inhibited the STAT3 and PTTG1 levels, and FAD with higher doses had stronger inhibitory effects (*p* < 0.05, Figures 4A,B). Then, the STAT3/PTTG1 protein level was detected by WB, and the results were consistent with those of qRT-PCR as FAD dose-dependently reduced p-STAT3 and PTTG1 expression (*p* < 0.05, Figure 4C). Finally, the immunoprecipitation (IP) experiment proved that STAT3 was bound to PTTG1 (Figure 4D). Therefore, FAD abated the STAT3/PTTG1 pathway in HCC cells.

### Inhibition of STAT3 Enhanced the Role of Falcarindiol in Promoting the Chemosensitivity of Hepatocellular Carcinoma

**FIGURE 5:**
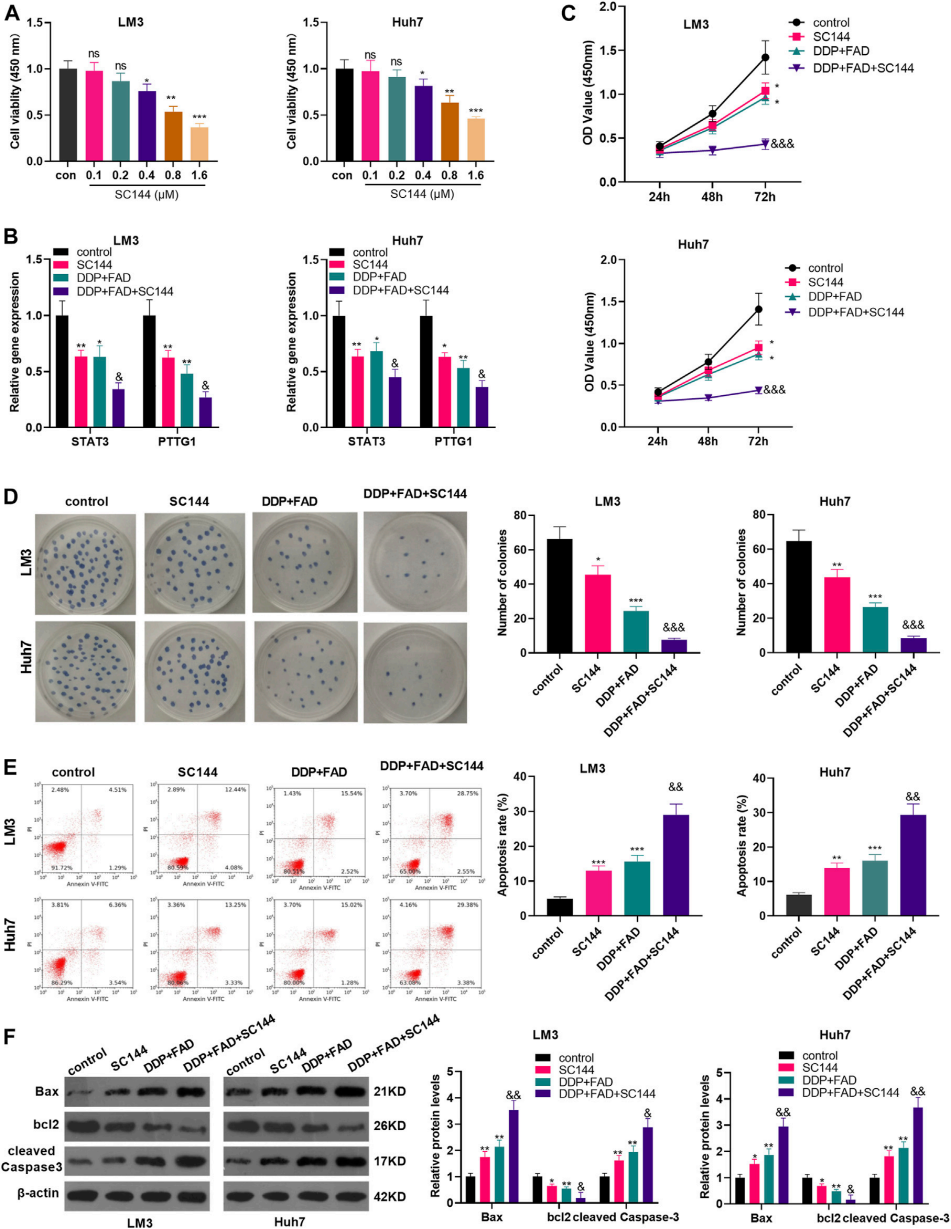
Inhibition of STAT3 strengthened the role of FAD-enhanced chemosensitivity of HCC cells to DDP. HCC cell lines (Huh7 and LM3) were treated with different doses of STAT3 inhibitor SC144 (0.1–1.6 µM) for 24 h. **(A)**. The cell proliferation was detected by CCK8 assay. HCC cell lines (Huh7 and LM3) were dealt with the DDP (2 μg/mL) and FAD (10 µM) and/or the STAT3 inhibitor SC144 (0.6 µM) for 24–72 h. **(B)**. qRT-PCR was used to detect the STAT3/PTTG1 expression. **(C, D)**. CCK-8 assay and colony formation assay was employed to test cell proliferation. **(E)**. FCM was conducted to monitor apoptosis. **(F)**. Expression of Bax, bcl2, cleaved Caspase3 was detected by WB. *p < 0.05, **p < 0.01 (vs. DDP group). & *p* < 0.05, && *p* < 0.01, &&& *p* < 0.001 (vs. DDP + FAD group). N = 3.

To confirm the role of STAT3 in FAD/DDP-mediated anti-tumor effects, the STAT3 inhibitor SC144 was administered in HCC cells. It was found that SC144 significantly reduced the proliferation of LM3 and Huh7 cells when its concentration was over 0.4 µM (Figure 5A). Next, the HCC cells were treated with SC144 (0.6 μM), or DDP (2 μg/mL) and FAD (10 µM). The qRT-PCR result suggested that SC144 reduced STAT3 and PTTG1 mRNA levels and further reduced STAT3/PTTG1 level in HCC cells treated with FAD/DDP (Figure 5B). Next, the CCK-8 assay, colony formation assay, and FCM testified that SC144 significantly repressed cell viability (time-dependently) and promoted apoptosis (vs. the control group) (Figures 5C–E). In addition, after being dealt with SC144, the cell proliferation was decreased and apoptosis was increased (compared with the DDP + FAD group) (*p* < 0.05 vs. FAD + DDP group, Figures 5C–E). WB showed that SC144 facilitated the expression of Bax and cleaved Caspase3 and abated the bcl2 level (compared with the DDP group). On this basis, the use of SC144 further strengthened the promotion on Bax and Caspase-3 and the inhibition on bcl2 (*p* < 0.05 vs. FAD + DDP group, Figure 5F). These results demonstrated that the inhibition of STAT3 enhanced the promoting effect of FAD/DDP on the chemosensitivity of HCC.

### Overexpressing PTTG1 Weakened the Falcarindiol-Enhanced DDP Chemosensitivity of Hepatocellular Carcinoma Cells

**FIGURE 6:**
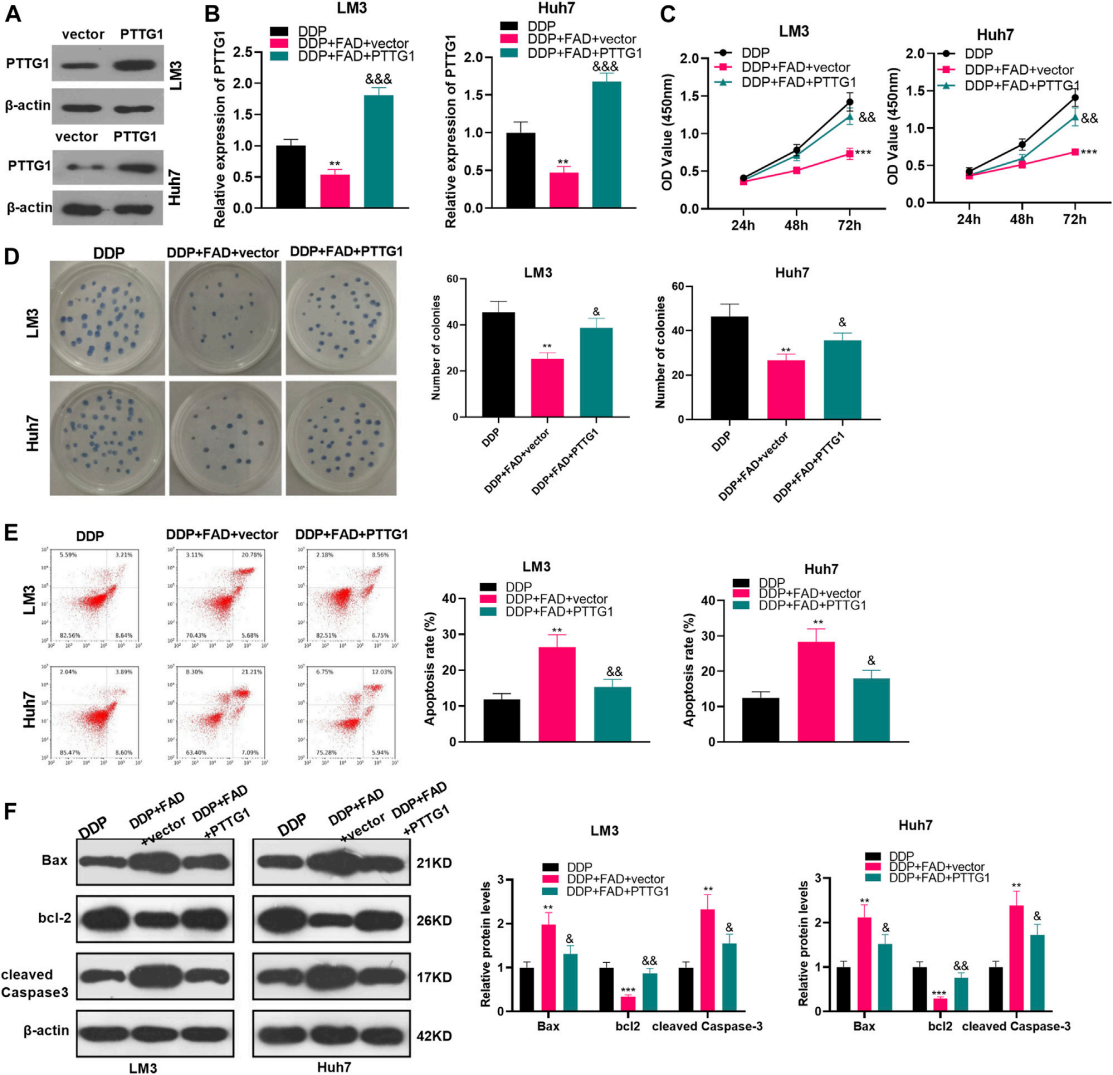
Overexpressing PTTG1 weakened the promotion of FAD-enhanced chemosensitivity of HCC cells to DDP. **(A)**. HCC cell lines (Huh7 and LM3) were transfected with PTTG1 overexpressing plasmids or negative vector. The protein level of PTTG1 was detected by WB. **(B)**. HCC cell lines (Huh7 and LM3) were treated with DDP (2 μg/mL) and FAD (10 µM) for 24 h. **(B)**. qRT-PCR was implemented to verify the STAT3/PTTG1 expression. **(C, D)**. CCK-8 assay and colony formation were conducted to detect cell proliferation. **(E)**. FCM was applied to test apoptosis. **(F)**. Expression of Bax, bcl2, and cleaved Caspase3 was detected by WB. **p < 0.01, ***p < 0.001 (vs. DDP group). & *p* < 0.05, && *p* < 0.01, &&& *p* < 0.001 (vs. the DDP + FAD + vector group). N = 3.

To clarify the role of PTTG1 in FAD-mediated effects, we constructed the PTTG1-overexpressed HCC cells (Figure 6A). Then the HCC cells were treated with DDP (2 μg/mL) and FAD (10 µM). In FAD + DDP-treated HCC cells, transfection of PTTG1 overexpression plasmids promoted PTTG1 mRNA expression (*p* < 0.05 vs. FAD + DDP + vector group, Figure 6B). The cell proliferation and apoptosis were detected by the CCK-8 assay, colony formation assay, and FCM, respectively. It turned out that overexpressing PTTG1 enhanced cell proliferation and inhibited cell apoptosis (*p* < 0.05, compared with the DDP + FAD + vector group) (*p* < 0.05, Figures 6C–E). WB was carried out to verify apoptosis, and the results manifested that Bax and cleaved Caspase3 were up-regulated, while bcl2 was down-regulated after overexpressing PTTG1 (*p* < 0.05, Figure 6F). The above results confirmed that overexpressing PTTG1 weakened the anti-tumor role of FAD on the chemosensitivity of HCC.

### Falcarindiol Facilitated the Chemosensitivity of Hepatocellular Carcinoma Cells to DDP in Nude Mice

**FIGURE 7:**
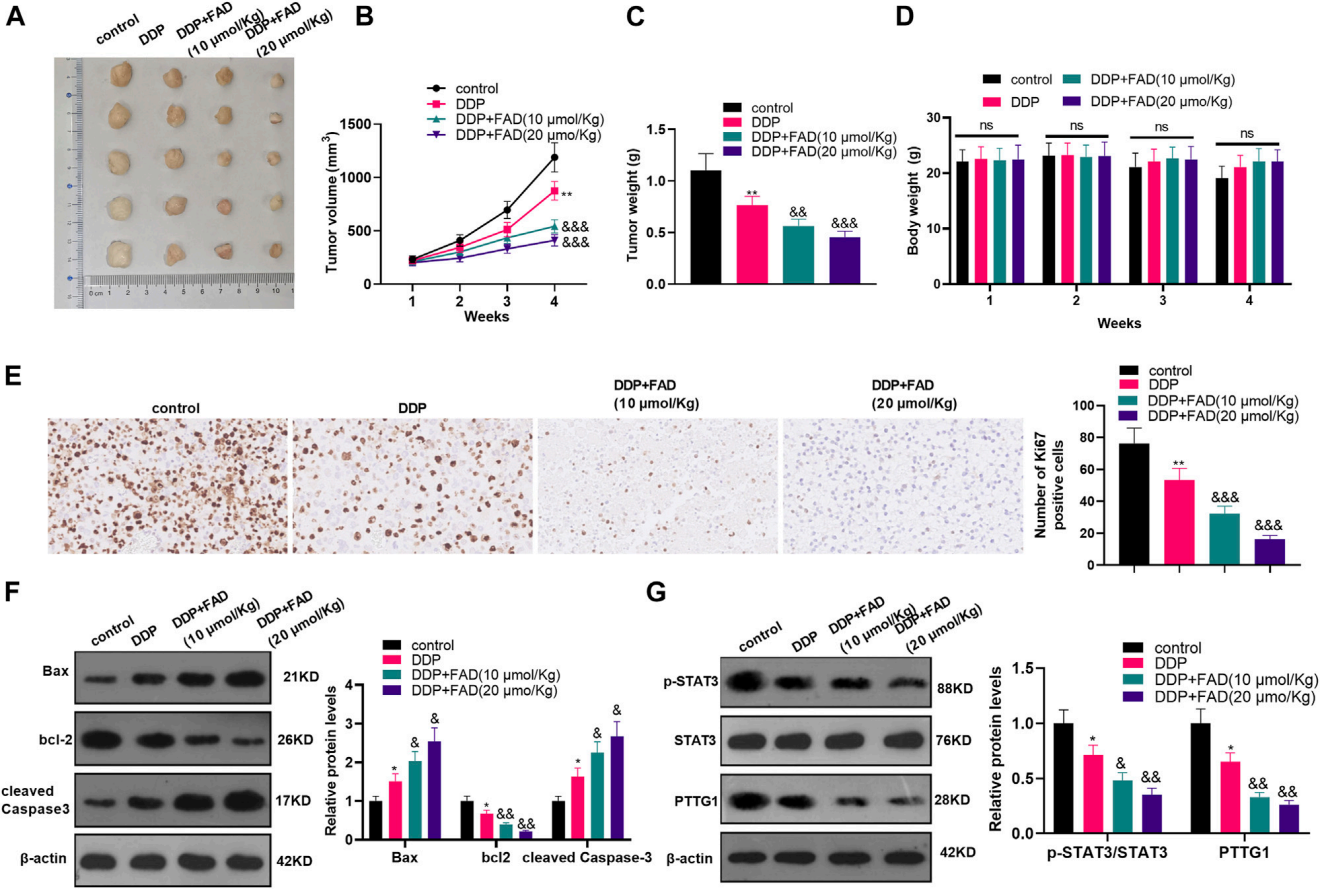
FAD promoted the chemosensitivity of HCC to DDP in nude mice. The nude mice were randomly divided into four groups. The mice were intraperitoneally injected with cisplatin (2 mg/kg) every 4 days for 28 days and/or FAD (10–20 mmol/kg) every other day for 28 days. On the 28th day, the nude mice were sacrificed and the tumor tissues were isolated. **(A)**. Tumor images were shown. **(B, C)**. The volume and weight of the mice removed tumors were calculated. **(D)**. The bodyweight of the nude mice was calculated. **(E)**. IHC was utilized to analyze the number of Ki67 positive cells. **(F, G)**. The expression of apoptosis-related proteins and STAT3/PTTG1 were compared by WB. *p < 0.05, **p < 0.01 (vs. DDP group). & *p* < 0.05, && *p* < 0.01, &&& *p* < 0.001 (vs. DDP + FAD group). N = 5.

We constructed a nude mouse model to explore the role of FAD *in vivo*. The tumor volume and weight were measured. The results showed that the DDP treatment decreased tumor weight and volume (*p* < 0.05, Figures 7A–C). The bodyweight of mice was calculated, and the data showed that both DDP and FAD treatment did not reduce the bodyweight of the nude mice (Figure 7D). With the additional treatment of FAD, the tumor volume and weight were attenuated compared with the DDP group (Figures 6A–C). We then conducted immunohistochemistry (IHC) to detect the Ki67 expression in the formed tumors. It was found that DDP attenuated the Ki67-positive cell rate, and FAD treatment further aggravated the loss of Ki67-positive cells (Figure 7E). Also, WB showed that the use of FAD on the basis of DDP treatment promoted the profiles of Bax and cleaved Caspase3 and inhibited the levels of bcl2, STAT3, and PTTG1 (*p* < 0.05, Figures 7F,G). The above results indicated that FAD facilitated the chemosensitivity of HCC to DDP *in vivo* through the inactivation of the STAT3/PTTG1 pathway.

## Discussion

In this study, the effect of FAD on the proliferation, apoptosis, and chemosensitivity of HCC cells and its regulatory mechanism was studied. Our experimental results demonstrated that FAD facilitated the chemosensitivity of HCC cells to DDP both *in vitro* and *in vivo*. Besides, FAD inhibited the PTTG1 expression by down-regulating STAT3, thus repressing HCC cell proliferation and promoting apoptosis.

Recently, the bioactive polyacetylenes isolated from apiaceous vegetables (including carrots, celery, celeriac, fennel, parsley, and parsnip) have been attracted increasing attention (Zidorn et al., 2005; Kramer et al., 2011). Among them, falcarinol and FAD, are both found to mediate biological processes, such as neural stem cell homeostasis (Kim et al., 2018), inflammatory response (Stefanson and Bakovic, 2020), lipid peroxidation (Ohnuma et al., 2011), granulocytic differentiation (Tsolmon et al., 2009), and cytotoxic activity (Cheung et al., 2019). Besides, several studies have found that FAD exerts tumor-suppressive effects by activating endoplasmic reticulum stress (Jin et al., 2012; Lu et al., 2017). Consistently, our findings revealed that FAD abated HCC cell proliferation and promoted apoptosis, thus enhancing the chemosensitivity of HCC to DDP.

Multiple mechanisms, such as epithelial-to-mesenchymal transition (EMT), angiogenesis, cancer stem-like cells (CSCs), immune microenvironment, were involved in chemoresistance in HCC treatment (Yamashita and Wang, 2013; Gurzu et al., 2019; Tao et al., 2019). Notably, many chemotherapeutic drugs repressed tumor cell growth and metastasis via inducing DNA damaging, which is dependent on repressing those oncogenes and signaling pathways that control DNA repair (Sakthivel and Hariharan, 2017). Among those DNA repair-related genes, Rad51, BRCA1, and MDC1 have been identified as vital genes. Overexpressed Rad51 (Harris et al., 2018), BRCA1 (Zhu et al., 2018), and MDC1 (Singh et al., 2020) contribute to genomic integrity and chemoresistance in cancer. Here, we also determined those genes in HCC cells treated with FAD or DDP. We found both FAD and DDP invention mitigated Rad51, BRCA1, and MDC1 expression. Interestingly, the combination of FAD and DDP led to more inhibitive effects on those factors’ expression. Therefore, we confirmed that FAD induced an anti-tumor role in HCC *via* repressing DNA repair.

Several studies have illustrated that STAT3 plays a carcinogenic effect on tumors. For instance, leptin stimulates prostate cancer cell proliferation and migration through STAT3 (Gorrab et al., 2020). In addition, KIF20A knockout reduces colorectal cancer cell proliferation and migration by inhibiting the JAK/STAT3 pathway (Zhang et al., 2020). On the other hand, some studies illustrated that the Hoveniae Semen Seu Fructus Ethanol extract exhibits anti-inflammatory activity *via* MAPK, AP-1, and STAT in lipopolysaccharide (LPS)-stimulated RAW 264.7 and mouse peritoneal macrophages (Jeong et al., 2019). Particularly, several studies have shown that dexmedetomidine induces AHSCs to secrete IL-6 by activating STAT3, thus expediting HCC evolvement (Chen et al., 2020). Other studies have found that Vitexin can be used as a STAT3 signal blocker to inhibit the survival and invasion of HCC cells (Lee et al., 2020). These results proved that targeting STAT3 is an effective method in HCC treatment. Interestingly, FAD mitigates the inflammatory response of murine macrophage RAW 264.7 cells induced by LPS by attenuating JNK, ERK, STAT1, and STAT3 (Venkatesan et al., 2018). Fortunately, in this study, we found that FAD repressed the STAT3 expression in HCC cells, and the use of SC144 further enhanced the promotion of FAD on DDP chemosensitivity of HCC.

PTTG1 is the transforming gene initially found in rat pituitary tumor cells. It has transcriptional activities and securin functions (Tong and Eigler, 2009) and also is a potential diagnostic biomarker in several cancers (Ren and Jin, 2017; Fraune et al., 2020; Ma et al., 2020). Recently, increasing studies have found that PTTG1 plays a central role in chemosensitivity, chromosome stability, and DNA repair (Vlotides et al., 2007). For instance, the deletion of PTTG1 in cells enhanced their doxorubicin sensitivity (Tong et al., 2011). While overexpression of PTTG1 increased senescence-associated (SA)-heterochromatin foci formation tumor cells, and that is p53-dependent and telomerase-independent (Hsu et al., 2010). In another study, downregulating PTTG1 results in a delay in centrosomal and noncentrosomal microtubule nucleation, and PTTG1 knockdown repressed cell polarization and migration (Moreno-Mateos et al., 2011). Here, the IP experiment showed that STAT3 could bind to PTTG1, and FAD inactivated the STAT3/PTTG1 pathway in HCC cells. Other studies manifested that the PTTG1 level was negatively correlated with the survival of HCC patients, so PTTG1 could be an independent prognostic biomarker for HCC (Lin et al., 2019). Moreover, some scholars have claimed that aconitum coreanum polysaccharide fraction (CACP) abates the growth of liver cancer cell H22 *in vitro* and *in vivo* by inhibiting PTTG1 (Liang et al., 2015). Consequently, the above studies suggest that PTTG1 is a vital regulator in chromosome stability and DNA repair in tumor cells, which include HCC cells. In the current study, we found that overexpressing PTTG1 elevated HCC cell proliferation and inhibited apoptosis. Furthermore, overexpressing PTTG1 weakened the suppression of FAD on the chemosensitivity of DDP of HCC.

In summary, our study found that FAD dampens HCC cell proliferation and promotes apoptosis by attenuating STAT3/PTTG1, which improves the chemosensitivity of HCC to DDP. Furthermore, the combined utilization of FAD with DDP effectively represses HCC tumor growth, suggesting that the combination of FAD with conventional cytotoxic drugs is a potential and effective therapeutic strategy for HCC. However, several limitations remain to be resolved in our future research: 1) whether FAD enhances chemosensitivity of the other drugs, such as oxaliplatin and 5-fluorouracil in HCC; 2) whether FAD displays a synergistic effect with those chemotherapeutic drugs *in vivo*; 3) the interaction between PTTG1 downregulation and DNA repair inhibition induced by FAD needs further exploration.

## Data Availability

The raw data supporting the conclusions of this article will be made available by the authors, without undue reservation, to any qualified researcher.
